# Fast genome-wide pedigree quantitative trait loci analysis using MENDEL

**DOI:** 10.1186/1753-6561-8-S1-S93

**Published:** 2014-06-17

**Authors:** Hua Zhou, Jin Zhou, Eric M Sobel, Kenneth Lange

**Affiliations:** 1 Department of Statistics, North Carolina State University, Raleigh, NC27695 USA; 2 Division of Epidemiology and Biostatistics, Mel and Enid Zuckerman College of Public Health, Tucson, AZ85721-0066, USA; 3 Department of Human Genetics, University of California, Los Angeles, CA90095, USA; 4 Department of Biomathematics, University of California, Los Angeles, CA90095, USA; 5 Department of Statistics, University of California, Los Angeles, CA90095, USA

## Abstract

The linkage era left a rich legacy of pedigree samples that can be used for modern genome-wide association sequencing (GWAS) or next-generation sequencing (NGS) studies. Family designs are naturally equipped to detect rare variants, control for population stratification, and facilitate the study of parent-of-origin effects. Unfortunately, pedigree likelihoods are notoriously hard to compute, and current software for association mapping in pedigrees is prohibitively slow in processing dense marker maps. In a recent release of the comprehensive genetic analysis software MENDEL, we implemented an ultra-fast score test for association mapping with pedigree-based GWAS or NGS study data. Our implementation (a) works for random sample data, pedigree data, or a mix of both;(b) allows for covariate adjustment, including correction for population stratification;(c) accommodates both univariate and multivariate quantitative traits; and (d) allows missing values in multivariate traits. In this paper, we assess the capabilities of MENDEL on the Genetic Analysis Workshop 18 sequencing data. For instance, when jointly testing the 4 longitudinally measured diastolic blood pressure traits, it takes MENDEL less than 51 minutes on a standard laptop computer to read, quality check, and analyze a data set with 959 individuals and 8.3 million single-nucleotide polymorphisms (SNPs). Our analysis reveals association of one SNP in the q32.2 region of chromosome 1. MENDEL is freely available on http://www.genetics.ucla.edu/software.

## Background

Pedigree data are attractive in modern association studies because they permit control of population substructure and study of parent-of-origin effects [1]. Related affecteds are also more likely to share the same disease-predisposing gene than unrelated affecteds. The classical variance component model has been a powerful tool for mapping quantitative trait loci in pedigrees [2].Polygenic effects are effectively captured by the kinship coefficient matrix as a variance component. In genome-wide association sequencing (GWAS), two alleles of a single nucleotide polymorphism (SNP) shift trait means and can be tested as a fixed effect. However, fitting a variance component model with pedigrees is computationally challenging, especially when it has to be done for a huge number of markers.We reexamine the computational bottlenecks and implement an ultra-fast score test when pedigree structure is explicitly given. Score tests require no additional iteration under the alternative model.All that is needed is evaluation of a quadratic form combining the score vector and the expected information matrix at the maximum likelihood estimates under the null model. Fast pedigree GWAS is now implemented in our software package MENDEL [3] for easy use by the genetics community. In this paper, we demonstrate the capabilities of MENDEL on the Genetic Analysis Workshop 18 (GAW18) sequencing data.

## Methods

Quantitative trait locus (QTL) association mapping typically invokes the multivariate Gaussian distribution to model the observed trait values $y=\left(y_{i}\right)$ over a pedigree. The standard model (2, Chapter 8) collects the corresponding means into a vector *ν *and the corresponding covariances into a matrix *Σ *and represents the loglikelihood of a pedigree as

$L=-\frac{1}{2}\;\text{lndet}\;\Sigma -\frac{1}{2}{\left(y-\nu \right)}^{t}\Sigma^{-1}\left(y-\nu \right),$

where the covariance matrix is typically parameterized as

(1) $$\Sigma =2\sigma_{a}^{2}\Phi +\sigma_{d}^{2}\Delta_{7}+\sigma_{h}^{2}H+\sigma_{e}^{2}I.$$

Here the variance component *Φ *is the global kinship coefficient matrix capturing additive polygenic effects, and $\Delta_{7}$ is a condensed identity coefficient matrix capturing dominance genetic effects. The household effect matrix *H *has entries $h_{ij}=1$ if individuals *i *and *j *are in the same household and 0 otherwise. Individual environmental contributions and trait measurement errors are incorporated via the identity matrix *i*. When one tests multiple traits, the covariance matrix has to be properly augmented by matrix Kronecker products. QTL fixed effects are captured through the mean component v=Aβ for some predictor matrix *A *and vector of regression coefficients *β*.

To implement likelihood ratio testing, iterative maximum likelihood estimation must be undertaken for each and every SNP under the alternative hypothesis. This unfortunate requirement is the major stumbling block retarding pedigree analysis. Score tests serve as convenient substitutes for likelihood ratio tests. A careful analysis shows that the basic elements of the score statistic can be quickly assembled. In MENDEL [3], SNPs with the most impressive score test *p*-values (top 50 by default) are further tested by the more accurate likelihood ratio method, thus achieving a good compromise of speed and power for large-scale QTL analysis.

## Results

### Data description

Our analysis is based on the genotype calls for 959 individuals (464 directly sequenced and the rest imputed) provided in the chrX-geno.csv.gz files. Simulated traits in all 200 replicates (SIMPHEN.1-200) were used for size and power studies in the first example. The second example presents results from a pedigree GWAS performed on chromosome 3 using the traits in the first simulation replicate (SIMPHEN.1). A whole genome QTL analysis for the real phenotype diastolic blood pressure (DBP) is presented in the final example.

### Adjustment for environmental effects

Both the traits (blood pressures) and some environmental factors are measured (or simulated) on study individuals at 3 or 4 visits. To adjust for the environmental effects of Age, BPMed, Smoke, and Sex, we model the systolic blood pressure (SBP) by a linear mixed model (LMM):

(2) $$SBP_{i,t}=\mu_{i}+Age_{i,t}\beta_{Age}+BPMed_{i,t}\beta_{BPMed}+Smoke_{i,t}\beta_{Smoke}+Sex_{i}\beta_{Sex}+\left(Age_{i,t}\times Sex_{i}\right)\beta_{Age\times Sex}+\varepsilon_{i,t},$$

where  i indexes individuals, *t *indexes 3 time points,  β s are the fixed effects, $\mu_{i}$ is an individual level random intercept assumed to be normal with covariance $cov\left(\mu_{i},\mu_{j}\right)=2\varphi_{ij}$, and $\varepsilon_{i,t}$ are independent standard normal errors. If we stack the traits $SBP_{i,t}$ into a column, this corresponds to a variance component model with a genetic component $2\sigma_{g}^{2}\left(1_{3}1_{3}^{t}⊗\Phi \right)$, where  Φ is the kinship coefficient matrix, and an environmental component $\sigma_{e}^{2}I_{3n}$. LMM is fitted by maximum likelihood (ML).

The estimated fixed effects for traits in simulation replicate 1 are summarized in Table 1. Estimates under the linear model (LM) are listed for comparison. Results from LMM imply significant additive genetic effects. The estimated heritability is 0.65 for SBP, 0.55 for DBP, and 0.63 for Q1. Residuals from LMM will be used as the multiple traits in QTL association mapping. Two types of residuals can be used. Residuals $r_{i,t}^{\left(1\right)}=SBP_{i,t}-\left({\hat{\mu }}_{i}+x_{i,t}^{t}\hat{\beta }\right)$, where ${\hat{\mu }}_{i}$ are the best linear unbiased estimate (BLUE) of the random intercept $\mu_{i}$, are *decorrelated *from the polygenic effects. QTL mapping can be performed on $r_{i,t}^{\left(1\right)}$ without the additive and dominant genetic components in (1). However, this strategy ignores the correlation between the longitudinal traits. Residuals $r_{i,t}^{\left(2\right)}=SBP_{i,t}-\left(\hat{\mu }+x_{i,t}^{t}\hat{\beta }\right)$, where $\hat{\mu }$ is the estimate for the grand intercept, yield the adjusted traits still containing the polygenic effects. QTL mapping using $r_{i,t}^{\left(2\right)}$ needs to keep the genetic components to properly capture the correlation between traits. In the following, we refer to the former as the decorrelated residuals (method 1) and to the latter as the correlated residuals (method 2).

**Table 1:** Summary of environmental effects for traits systolic blood pressure(top), diastolic blood pressure(middle) and Q1 (bottom) in simulation replicate SIMPHEN.1

SBP	*μ*	$\beta_{Age}$	$\beta_{BPMed}$	$\beta_{Smoke}$	$\beta_{Sex}$	$\beta_{Age\times Sex}$	$\sigma_{g}^{2}$	$\sigma_{e}^{2}$	$R^{2}$
LM LMM	119.360 (0) 119.739 (0)	0.135 (2 × 10^−11^) 0.168 (1 × 10^−11^)	13.088 (7 × 10^−91^) 6.981 (0)	0.284 (6 × 10^−1^) 0.556 (4 × 10^−1^)	−19.547 (1 × 10^−49^) −20.985 (0)	0.387 (4 × 10^−43^) 0.418 (0)	-- 112.58	139.558 58.128	42.4% 74.46%

**DBP**	μ	$\beta_{Age}$	$\beta_{BPMed}$	$\beta_{Smoke}$	$\beta_{Sex}$	$\beta_{Age\times Sex}$	$\sigma_{g}^{2}$	$\sigma_{e}^{2}$	$R^{2}$

LM LMM	75.781 (0) 75.382 (0)	−0.052 (7 × 10^−4^) −0.032 (8 × 10^−2^)	1.893 (7 × 10^−5^) −0.751 (1 × 10^−1^)	−0.109 (8 × 10^−1^) −0.087 (9 × 10^−1^)	−8.201 (2 × 10^−16^) −8.305 (7 × 10^−12^)	0.124 (5 × 10^−9^) 0.131 (3 × 10^−7^)	-- 49.848	81.632 40.395	4.8% 54.8%

**Q1**	μ	$\beta_{Age}$	$\beta_{BPMed}$	$\beta_{Smoke}$	$\beta_{Sex}$	$\beta_{Age\times Sex}$	$\sigma_{g}^{2}$	$\sigma_{e}^{2}$	$R^{2}$

LM LMM	38.642 (0) 39.115 (0)	−0.087 (2 × 10^−3^) −0.079 (1 × 10^−3^)	−2.508 (3 × 10^−2^) −2.211 (3 × 10^−2^)	0.270 (7 × 10^−1^) 0.239 (7 × 10^−1^)	8.904 (4 × 10^−8^) 8.809 (1 × 10^−9^)	0.005 (9 × 10^−1^) 0.000 (9 × 10^−1^)	-- 53.373	85.260 31.615	21.9% 76.8%

Numbers in parenthesis are *p*-values.

*DBP*, diastolic blood pressure; *SBP*, systolic blood pressure.

### Size and power study (using SIMPHEN.1-200)

**Figure 1:**
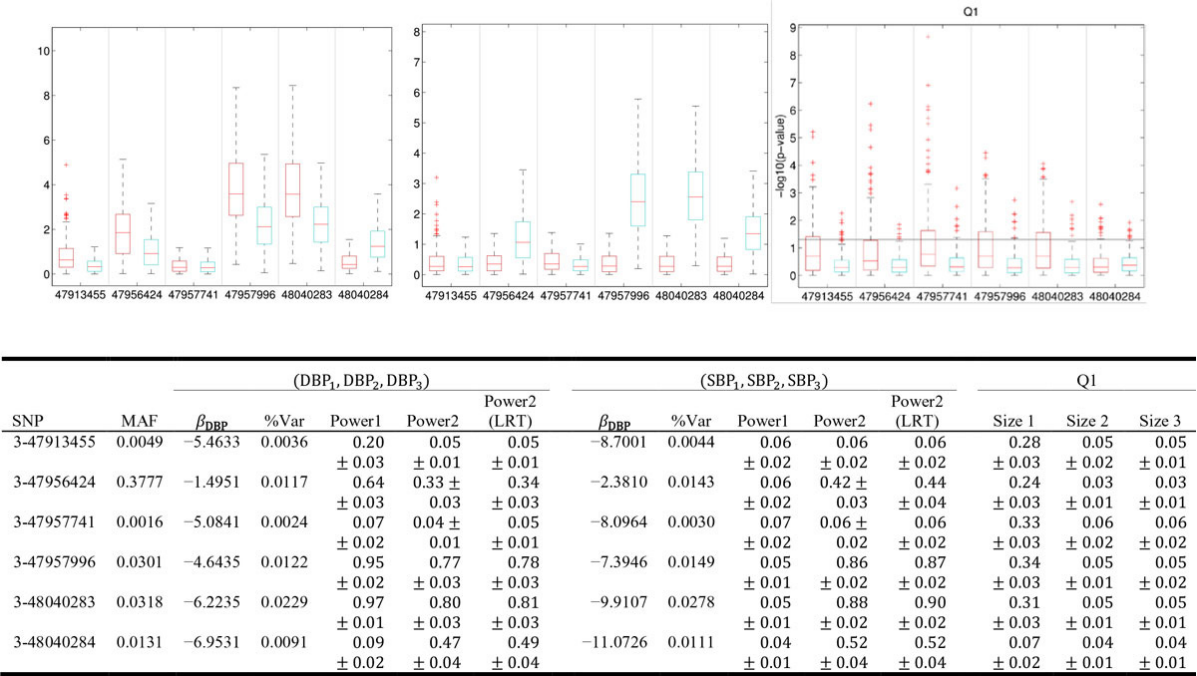
**Results of power and size study**. Top: Box plots of −log10(*p*-values) from score tests for the 6 functional variants in MAP4 based on 200 simulation replicates. The red (left) ones use the decorrelated residuals (method 1). The blue (right) ones use the correlated residuals (method 2). The horizontal line represents the 0.05 significance level. Bottom: Empirical power and type I error.

Powers for detecting the 6 functional variants in the *MAP4 *gene on chromosome 3 are evaluated based on the provided 200 simulation replicates. Figure 1 displays the box plots of the 200 $-log_{10}$(*p*-values) for each variant using either the decorrelated (method1) or the correlated residuals (method2). Type I errors are evaluated based on the provided Q1 trait which is not genetically influenced. In general, we found that the decorrelated residuals (method1) lead to higher power but also inflated type I error. The test using the correlated residuals (method2) has well-controlled type I error, high power (0.78 *~ *0.90) for detecting the common variants 47957996 and 48040283 but low power for the rare variants 47913455 and 47957741. For comparison, we also list the power and the size of likelihood ratio test (LRT) using correlated residuals. LRT edges out the score test in a few cases, but the difference is not significant. LRT is practically infeasible for a large number of SNPs. In the following two pedigree GWAS examples, we present only the results of the score test using correlated residuals (method 2).

### Pedigree Genetic Analysis Workshopon chromosome 3 (using SIMPHEN.1)

**Figure 2:**
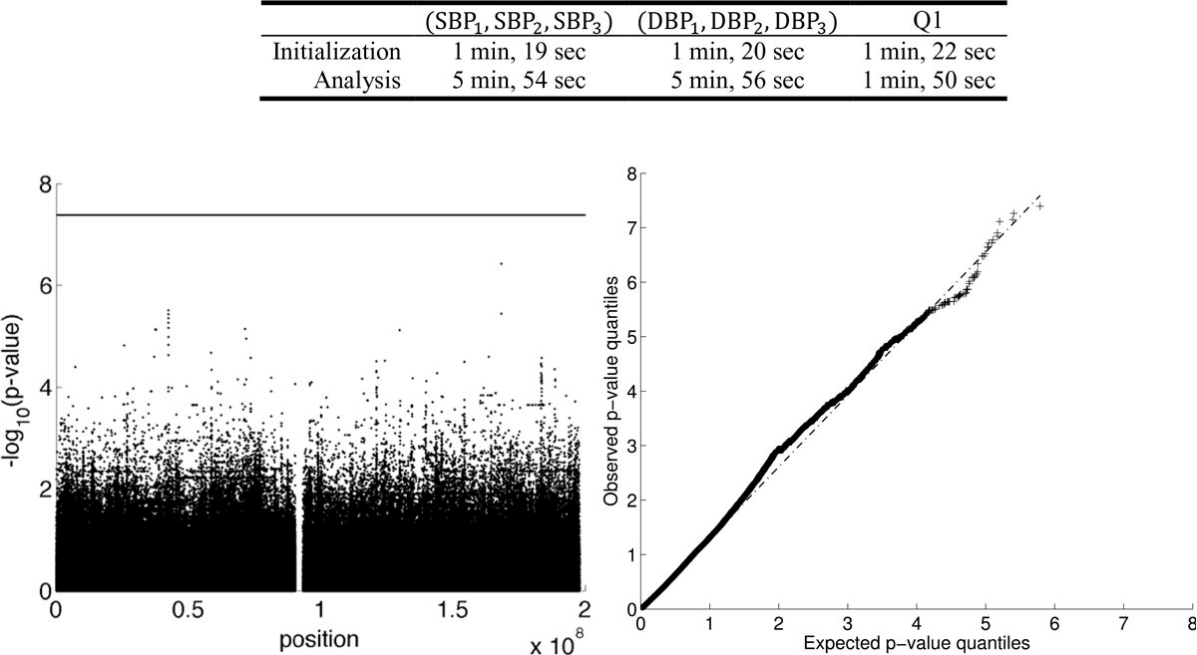
**Results of pedigree genome-wide association sequencing for testing traits systolic blood pressure (SBP), diastolic blood pressure (DBP) and Q1 in simulation replicate SIMPHEN.1 on the 1,213,657 single-nucleotide polymorphisms on chromosome 3 and 849 individuals. **Top: Run times on a standard laptop. Bottom: Manhattan plot (left) and QQ plot (right) for the traits $\left(DBP_{1},DBP_{2},DBP_{3}\right)$. The horizontal line represents the genome-wide significance level. Plots for SBP and Q1 are similar and are omitted here.

We performed pedigree GWAS on all available sequence variants on chromosome 3 using the correlated residuals from the traits in SIMPHEN.1.A total of 1,213,657 SNPs passed the filtering and were subject to testing.Figure 2 displays the run times and the Manhattan and quartile-quartile(QQ) plots for jointly testing the multivariate traits SPB. No variants passed the genome-wide significance level (horizontal line). For the null trait Q1, 5.29% of SNPs have *p*-values less than 0.05, corroborating the correct size of the score test. Results for trait SBP are similar and not displayed.

### Analysis of real phenotypes diastolic blood pressure

The phenotypes (SBP and DBP measured at 4 time points) are available for 1389 members from 20 extended families. The largest family contains 107 individuals; the smallest, 27. Genotypes at 8,348,674 SNPs were available on 959 of the individuals. For brevity, we only present results for the multivariate DBP trait here.

**Figure 3:**
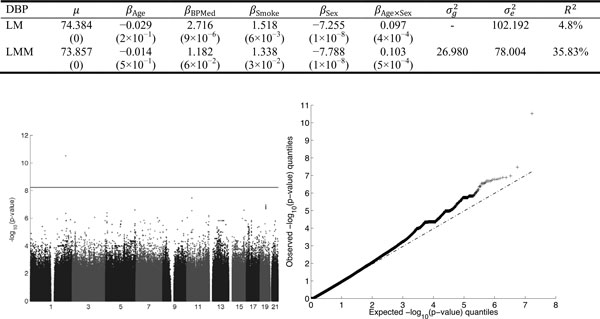
**Results for pedigree genome-wide association sequencing of 8,348,674 single-nucleotide polymorphisms for the real diastolic blood pressure (DBP) traits**. Top: Environmental effects fitted from linear model (LM) and linear mixed model (LMM). Numbers in parenthesis are *p*-values. Bottom: Manhattan plot (left) and quartile-quartileplot (right). The horizontal line represents the genome-wide significance level.

We adopted the strategy discussed earlierto adjust the multivariate traits for the environmental factors. The table in Figure 3 summarizes the effects of environmental effects estimated by LM and LMM (2). The estimated heritability of the DBP traits is 0.2564. We analyzed all SNPs and pedigrees together for the multivariate traits $\left(DBP_{1},DBP_{2},DBP_{3},DBP_{4}\right)$. To read in all the data and run standard QC procedures took 10 minutes and 14 seconds. This QC excluded 10,603 SNPs and 124 individuals based on genotyping success rates below 98%. The subsequent ped-GWAS analysis ran in 40 minutes and 55 seconds and included all of the results plotted in Figures 3. The complete run never used more than 3.2 GB of RAM.

The most significant *p*-value found by whole genome analysis was 1 × 10^−10.5 ^on chromosome 1 q32.2 region at 210,338,112 base pairs. No other SNPs reached genome-wide significance.

## Conclusions

By supplying a comprehensive, fast, and easy-to-use package for GWAS on quantitative traits in general pedigrees, we hope to encourage exploitation of family-based data sets for gene mapping. A gene mapping study should collect as large a sample as possible consistent with economic constraints and consistent trait phenotyping.If the sample includes pedigrees, all the better. Here we have argued that score tests can efficiently handle unrelated individuals, pedigrees, or a mixture. For human studies, in whichcontrolling breeding is forbidden, nature has provided pedigrees segregating every conceivable genetic trait. Many of these pedigrees are known from previous linkage studies and should be treasured as valuable resources.

## Competing interests

The authors declare that they have no competing interests.

## Authors' contributions

HZ, EMS and KL designed the overall study. HZ and JZ conducted statistical analyses and drafted the manuscript. All authors read and approved the final manuscript.
